# QTL mapping for adult plant resistance to wheat stripe rust in M96-5 × Guixie 3 wheat population

**DOI:** 10.1007/s13353-022-00686-z

**Published:** 2022-03-26

**Authors:** Bin Cheng, Xu Gao, Ning Cao, Yanqing Ding, Tianqing Chen, Qiang Zhou, Yu Gao, Zhihai Xin, Liyi Zhang

**Affiliations:** 1 Guizhou Institute of Upland Crops, Guizhou Academy of Agricultural Sciences, Guiyang, 550006 Guizhou People’s Republic of China; 2 Chengdu Institute of Biology, Chinese Academy of Sciences, Chengdu, 610041 Sichuan People’s Republic of China; 3 College of Agriculture, Guizhou University, Guiyang, 550025 Guizhou People’s Republic of China; 4 College of Life Sciences, Guizhou University, Guiyang, 550025 Guizhou People’s Republic of China

**Keywords:** Common wheat, *Puccinia striiformis* f*.* sp. *tritici* (*Pst*), Adult plant resistance, SNP array, Linkage analysis

## Abstract

Development of cultivars with multiple resistances has proven to be an effective way to prevent diseases in wheat breeding. The Guixie 3 variety (GX3) has shown excellent performance in resistance to stripe rust in field for many years. The purpose of this study was to detect quantitative trait loci (QTL) associated with resistance to stripe rust in the adult plant stage and determine closely linked molecular markers. A population of recombinant inbred lines (*n* = 228) was derived from a cross between the susceptible landrace Mian 96–5 (M96-5) and GX3 variety and evaluated in multiple field studies, and QTL analysis enabled to elucidate genetic architecture of wheat resistance to stripe rust. A total of 19 QTL for stripe rust resistance were mapped on 12 chromosomes using phenotypic data from multiple field tests over the course of 6 years. These chromosomes included 1B (2), 1D (2), 2A (2), 2B (2), 2D (1), 4B (2), 4D (1), 5A (3), 5B (1), 6A (1), 6B (1), and 7B (1). Two stable QTL on chromosomes 2AS (*Qyr.gaas.2A*) and 6AL (*Qyr.gaas.6A*) were detected in six and five different environments, respectively; in both QTL, positive allele was contributed by GX3 variety. *Qyr.gaas.2A* was found to be crucial for increasing adult plant resistance, which may explain the large phenotypic variation of 45.52%. Our results provide theoretical and molecular insight for wheat breeding and suggest the cloning of genes associated with the GX3 variety may be beneficial in future studies.

**Supplementary Information:**

The online version contains supplementary material available at 10.1007/s13353-022-00686-z.

## Introduction

Wheat stripe rust, caused by *Puccinia striiformis* f. sp. *tritici* (*Pst*), is one of the most damaging diseases associated with global wheat production (Wellings 2011). Since 1949, there have been four epidemics of wheat stripe rust in China (1950, 1964, 1990, and 2002), resulting in a loss of more than one million tons of wheat per year (Kang et al. 2015). The effective use of disease-resistant varieties is crucial for the control of wheat stripe rust (Bai et al. 2014). High-yield varieties with resistance to stripe rust are environmentally friendly (Elshafei et al. 2021). There are two main types of genetic wheat resistance to stripe rust. One is a resistance that presents itself at the seedling stage (or all-stage resistance [ASR]); this form of resistance is generally effective during the whole growth period. The second is adult plant resistance (APR), which usually provides partial resistance to all races at post-seedling stages.

Epidemics are caused by a loss of effective resistance genes against stripe rust in wheat production (Han et al. 2015). Currently, wheat varieties carrying the stripe rust resistance gene *Yr24/Yr26* that were once widely used in China (Hu et al. 2014), such as Chuanmai 42 (Liu et al. 2010) and Guinong 22 (He et al. 2011), have lost their resistance due to the emergence of a pathogenic group (V26). At the National Wheat Rust and Powdery Mildew Research Collaborative Group Meeting, which took place in China in 2016, the pathogenic group (V26) of Guinong 22 was officially named Chinese yellow rust 34 (CYR34). At present, among the 83 (*Yr1*–*Yr78*) officially designated resistance genes and 47 proposed resistance genes (Maccaferri et al. 2015; McIntosh et al. 2017), only a few seedling disease resistance (ASR) genes (*Yr5*, *Yr15*, *Yr53*, *Yr61*, *Yr64*, *Yr65*, and *Yr69*) and adult plant disease resistance genes (APR) (*Yr18*, *Yr30*, *Yr32*, *Yr36*, *Yr39*, *Yr52*, *Yr54*, *Yr59*, and *Yr62*) still maintain effective resistance to wheat stripe rust (Hou et al. 2016; Lu et al. 2014; Zeng et al. 2015; Zhou et al. 2015). Therefore, to ensure the sustainable management of wheat stripe rust in southwest China, it is imperative to discover new stripe rust resistance genes, to identify resistance-associated molecular markers, and to ultimately breed new disease-resistant wheat varieties.

In recent years, with the development of high-throughput genotyping technology, single-nucleotide polymorphism (SNP) arrays have been widely used in wheat. An example includes the construction of a high-density genetic map with stripe rust resistance gene/quantitative trait locus (QTL) mapping (Chen et al. 2016a; Gao et al. 2016; Jighly et al. 2015; Liu et al. 2015; Winfield et al. 2015; Wu et al. 2018a, 2018b) and genome-wide association analysis (Kertho et al. 2015; Liu et al. 2017; Naruoka et al. 2015; Zegeye et al. 2014). The wheat 55 K SNP array is an economical medium-density SNP chip developed from the wheat 660 K SNP array (Jia and Zhao 2016) and has been used in many different studies. The 660 K SNP array has been used to provide a genetic map of the *P* genome of *Agropyron* (Zhou et al. 2018), to identify the gene for grain weight using an integrated genetic map with > 100,000 SNPs (Cui et al. 2017), and to map QTL for stripe rust resistance in adult stage of wheat (Wu et al. 2017).

Wheat wild relatives can be used as a resource bank of disease resistance genes. Some genes have been officially classified for stripe rust resistance (Maccaferri et al. 2015). Wild emmer wheat (*T*. *dicoccoides*), a wild tetraploid ancestor of common wheat, has good resistance for wheat stripe rust and importantly shows great potential for wheat breeding. A number of resistant genes have been previously identified in wild emmer wheat, such as *YrH52*, *Yr15*, *Yr35*, and *Yr36* (Li et al. 2008; Peng et al. 1999; Uauy et al. 2005; Wang et al. 2018). Oat belongs to the *Gramineae Aveneae Dumort Avena* L. variety and has good resistance to biotic (wheat rust and scab) and abiotic (drought, cold, and barren) stress (Han et al. 2008; Sharma and Gill 1983; Zhang 1999). GX3 was obtained by distant hybridization of wild emmer wheat (*T*. *dicoccoides*) with wild oat (*Avena fatua* L. var. *glabrata pat*) and then backcrossed with common wheat (Guinong 22). It has shown resistance to the current wheat stripe rust epidemic for many years. In this study, we use a wheat 55 K SNP array to map QTL for APR to stripe rust employing recombinant inbred line (RIL) population of “M96-5/GX3,” to identify tightly linked molecular markers for their use in future marker-assisted breeding.

## Materials and methods

### Plant materials

The susceptible winter landrace line M96-5 and the resistant line GX3 were used as the parental lines for this study. The mapping population comprised of 228 F_6:7_ RILs from crossing M96-5 × GX3. The GX3 line is a semi-winter, late maturity, long spikelet variety of common wheat, and its entire growth period is an estimated 210 days. The susceptible line M96-5 has large spikelet with excellent agronomic traits, and its growth period is an estimated 190 days. The RIL population was established by Dr. Zhou Qiang from the Chengdu Institute of Biology, Chinese Academy of Sciences (Chengdu, Sichuan Province). Avocet S (AVS) and SY95-71 were used as susceptible controls throughout the study.

### Phenotyping

In these field trials, two parents and a RIL population were used to test resistance in mixed races of stripe rust within a natural setting. These were planted in October 2016 in Mianyang, Sichuan Province (MY17, 31° 23′ N, 104° 49′ E, altitude 478 m); in October 2017, 2018, and 2019 in Guiyang, Guizhou Province (GY18, GY19, and GY20, 26° 29′ N, 106° 39′ E, altitude 1175 m); and in November 2019 in Anshun, Guizhou Province (AS20, 26° 24′ N, 105° 96′ E, altitude 1280 m) and Shuangliu, Sichuan Province (SL20, 30° 57′ N, 103° 92′ E, altitude 498 m). During planting season (October of the sowing year — May of harvest year) in different environments, the total rainfall of MY17 was 265.08 mm, and the average temperature was 13.53 °C, GY18 was 611.35 mm/12.17 °C, GY19 was 736.97 mm/10.94 °C, GY20 was 730.40 mm/12.24 °C, AS20 was 785.64 mm/13.01 °C, and SL20 was 333.52 mm/13.97 °C (for specific information, please see Table S4). We fertilized 450 kg compound fertilizer (N + P_2_O_5_ + K_2_O ≥ 45%) per hectare in the field before planting wheat. The ratio of nitrogen, phosphorus, and potassium is 19–15-11 in the compound fertilizer.

There were two rows per line with two replications, 30 seeds per row of 1 m, 10 lines per block, and the susceptible line AVS (or SY95-71) was planted every five lines as the control line. The first severe degree was recorded when the disease severity of AVS (or SY95-71) in the control group and the susceptible parent M96-5 reached 50% or more (i.e., the area of rust fungus accounted for more than half of the entire leaf). In accordance with the percentage of the total leaf area occupied by rust fungus, the severity of stripe rust was also recorded visually for each wheat family. More than 5 individual plants were observed in each row, and data of each row was the overall average. Recordings were taken every other week until the susceptible control reached 100%, known as the maximum disease severity (MDS), and got the MDS data at each environment (two replications × one MDS data × six environments). The modified Cobb scale was referred to for the phenotypic data required for QTL analysis (i.e., 1, 5, 10, 20, 30, 50, 60, 80, and 100%) (Li and Zeng 2002).

### Statistical analysis

To estimate the genetic and environmental effects in each line, we compared the environments and line × environment interactions using the AOV function in IciMapping 4.1 software (Zeng et al. 2019), and significance was measured by analysis of variance (ANOVA). The formula of broad sense heritability $h_b^2=\sigma_g^2/(\sigma_g^2+\sigma_g^2/e+\sigma_g^2/re)$, where $\sigma_g^2$ is (MS_f_ − MS_fe_)/*re*, $\sigma_{ge}^2$ is (MS_fe_ − MS_e_)/*r*, and $\sigma_\varepsilon^2$ is MS_e_, $\sigma_g^2$ = genetic variance, $\sigma_{ge}^2$ = genotype × environment interaction variance, $\sigma_{ge}^2$ = error variance, MS_f_ = mean square of genotypes, MS_fe_ = mean square of genotype × environment interaction, MS_e_ = mean square of error, *r* = number of replications, and *e* = number of environments. The correlation between multiple field conditions was analyzed by the *Pearson* method in SPSS v20 software.

### Genotyping

Genomic DNA was extracted using the cetyltrimethylammonium bromide (CTAB) method (Saghai-Maroof et al. 1984). Two parents and a RIL population were genotyped using the 55 K SNP array by China Golden Marker (Beijing) Co., Ltd. (http://www.cgmb.com.cn/). Basic quality control (QC) tests were performed on samples by measuring markers based on genotype data detection rate, minimum allele frequency (MAF), and heterozygosity. The criteria used for sample quality control were as follows: DQC > 0.82, detection rate ≥ 85%, and heterozygosity rate ≤ 10%; the criteria for marking quality control were as follows: detection rate greater than or equal to 95%, and MAF of 5% or more, heterozygosity rate of 50% or less, and the number of alleles was 2.

Based on the preliminary results of QTL mapping, 22 pairs of simple sequence repeat (SSR) markers located on the 2AS chromosome were selected for genetic map construction (see Table S1). These markers were identified from GrainGenes (https://wheat.pw.usda.gov/GG3/) (Somers et al. 2004), and PCR reactions and polyacrylamide electrophoresis were performed as previously reported (Wu et al. 2018a). To distinguish the difference between *Yr17* and major QTL on chromosome 2AS identified in this study, the specific CAPS marker *URIC/LN2* for *Yr17* was used to scan wheat lines with GX3 pedigree and the carrier line of *Yr17* gene (VPMI) followed by digesting of restriction enzyme *Dpn*II. Detecting procedure for *URIC/LN2* was processed according to previously report (Helguera et al. 2003).

### Map construction and QTL analysis

The BIN function in QTL IciMapping v4.1 software (http://www.isbreeding.net/) (Meng et al. 2015) was used for redundant marker screening. In the mapping software, JoinMap v4.0, the LOD (Likelihood of odd) value was set to 3.0 for linkage analysis, and genetic map distance was calculated using the Kosambi function. QTL analysis was performed using QTL IciMapping v4.1 software, and the LOD threshold was set to 2.5. Mapchart (https://www.wur.nl/en/show/Mapchart.htm) was used to draw images (Voorrips 2002). The R package R/qtl was used to draw the genetic map (Broman et al. 2003).

### Prediction of candidate genes

In order to get the physical location of the polymorphic SNP marker, the SNP probes of these markers were aligned with respect to the released Chinese Spring sequence through a BLAST search (Reference Sequence v1.0, the International Wheat Genome Consortium (IWGSC), http://www.wheatgenome.org/). The specific parameters of blastn are default as follows: expect threshold was 0.001, word size was 15, max target sequence was 50, match/mismatch scores were 2 or − 3, gap costs were existence: 5 and extension: 2, at the same time, excluding low complexity region.

We could get information of gene annotation conveniently from this website: (http://202.194.139.32); the Triticeae Multi-omics Center provides genome, transcriptome, proteome, and epigenome dataset resources for common wheat and relatives as well as useful tools such as the Basic Local Alignment Search Tool (BLAST), sequence extraction, and the design of molecular markers and primers. It also aligned the flanking sequences of the Wheat 55 K SNP array to the Chinese Spring reference genome V1.0. Based on the alignment results in the Triticeae Multi-omics Center, unique probes with reliable physical positions in the 55 K SNP array were extracted (Sun et al. 2020). We got the information of candidate genes with annotations in this browser based on the beginning and ending physical position of the marker interval, e.g., chr2A:16618124..31858124.

## Results

### Evaluation of resistance

**Fig. 1:**
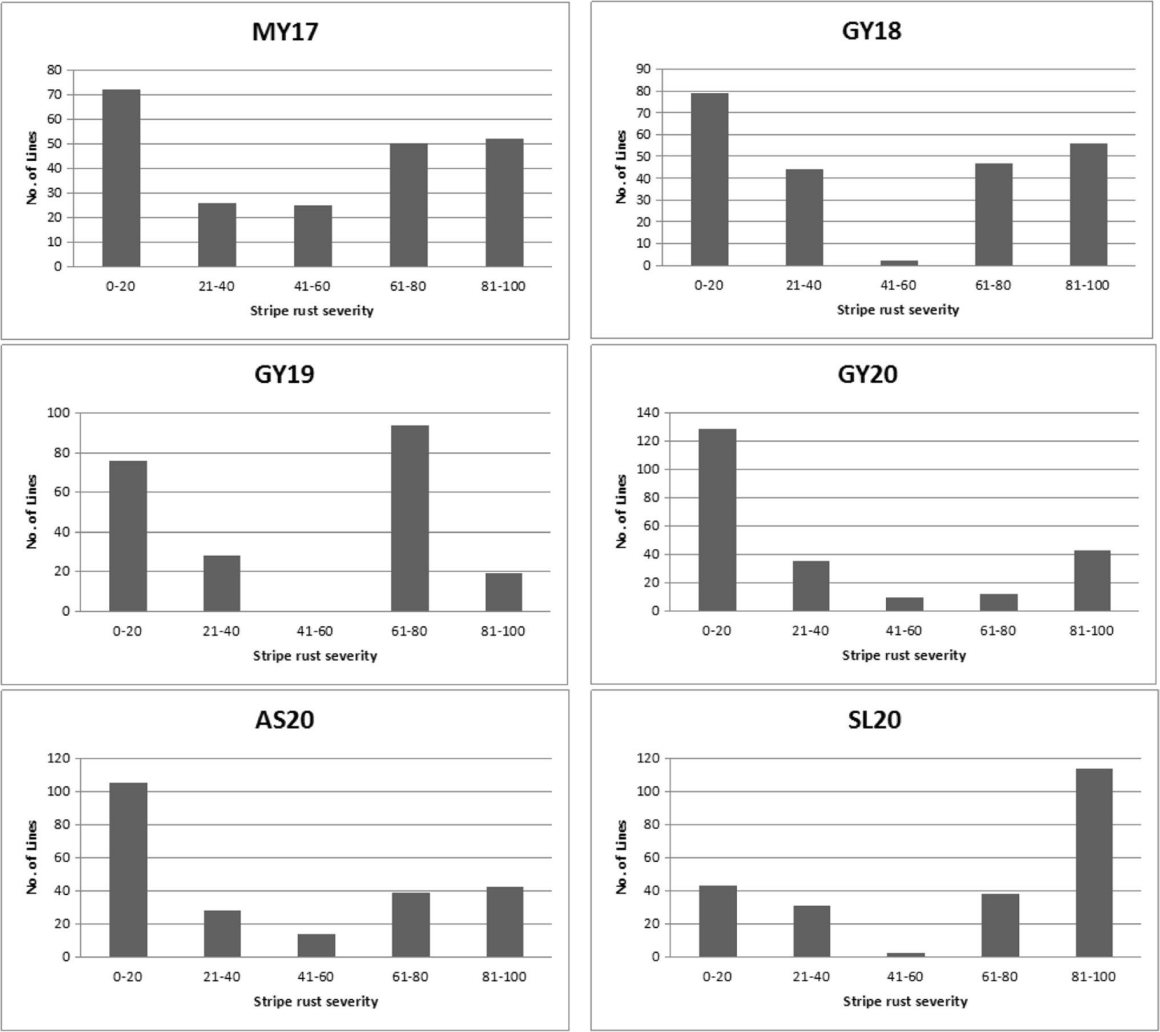
Frequency distributions of stripe rust maximum disease severities (MDS) in the 228 RIL population derived from a cross between the susceptible landrace M96-5 and the resistant variety GX3 across six environments: MY17, 2017 Mianyang; GY18, 2018 Guiyang; GY19, 2019 Guiyang; GY20, 2020 Guiyang; AS20, 2020 Anshun; SL20, 2020 Shuangliu

**Fig. 2:**
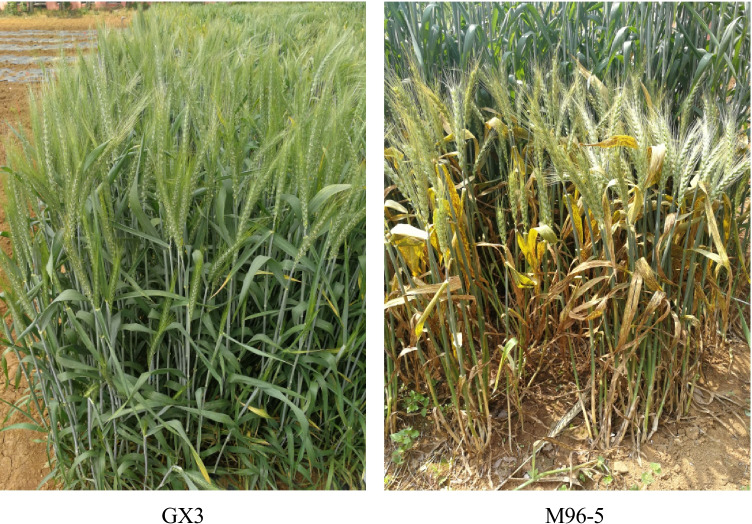
The phenotype of the parent material in the field at the adult stage

In the six different field trials, phenotypic data indicated significant genetic variation in APR. M96-5 and GX3 obtained mean MDS scores of 100% and 0%, respectively. MDS of the mapping populations ranged from 0 to 100% in each field study, and the phenotypic data were not continuously distributed (Figs. 1, 2). Through the Kolmogorov–Smirnov test, the bilateral significance of the phenotype data from all environments was less than 0.10; the data does not follow a normal distribution. The phenotype data indicated that the separation rate of resistant lines and susceptible lines was close to 1:1. *Pearson*’s correlation among the six field trials ranged from 0.38 to 0.79 (*P* < 0.001) (Table 2). ANOVA analysis including replicates for each experiment showed that there was significant variation in MDS when comparing different locations and wheat lines. Wheat lines combined with environment interactions also differed significantly, and the heritability between different locations was also found to be significantly high (0.91). These results indicated that the QTL in APR had a dramatic effect in decreasing disease severity (Table 1, 2).

**Table 1:** Variance components of disease severity (DS) scores for the 228 RIL population derived from M96-5 × GX3 across six environments

Source of variation	*Df*	Mean square	*F* value
RILs	227	8911.40	272.59**
Replicates/environment	6	23,823.53	728.74**
Environments	5	56,632.48	1732.34**
Lines × environment	1121	872.79	26.70**
Error	1348	32.69	
${h}_{b}^{2}$	0.91		

^**^Significance at *P* < 0.01

**Table 2:** Correlation analysis (*r*) of disease severity (DS) of the M96-5 × GX3 RIL population across six environments

Environment	MY17	GY18	GY19	GY20	AS20	SL20
MY17	1					
GY18	0.75***	1				
GY19	0.58***	0.69***	1			
GY20	0.65***	0.77***	0.60***	1		
AS20	0.66***	0.67***	0.60***	0.79***	1	
SL20	0.60***	0.50***	0.38***	0.43***	0.48***	1

^***^Significance at *P* < 0.001. MY17, 2017 Mianyang; GY18, 2018 Guiyang; GY19, 2019 Guiyang; GY20, 2020 Guiyang; AS20, 2020 Anshun; SL20, 2020 Shuangliu

### Construction of genetic linkage map

Whole genome analysis of the two parental lines and 228 RILs was performed using the wheat 55 K SNP array. Within the 55,000 SNPs, 7570 were identified as polymorphic markers to distinguish between M96-5 and GX3. A total of 589 were excluded because they had missing data (> 10%) or showed segregation distortion. The remaining 6981 SNPs fell into 1543 bins, and 5438 SNPs were excluded.

**Fig. 3:**
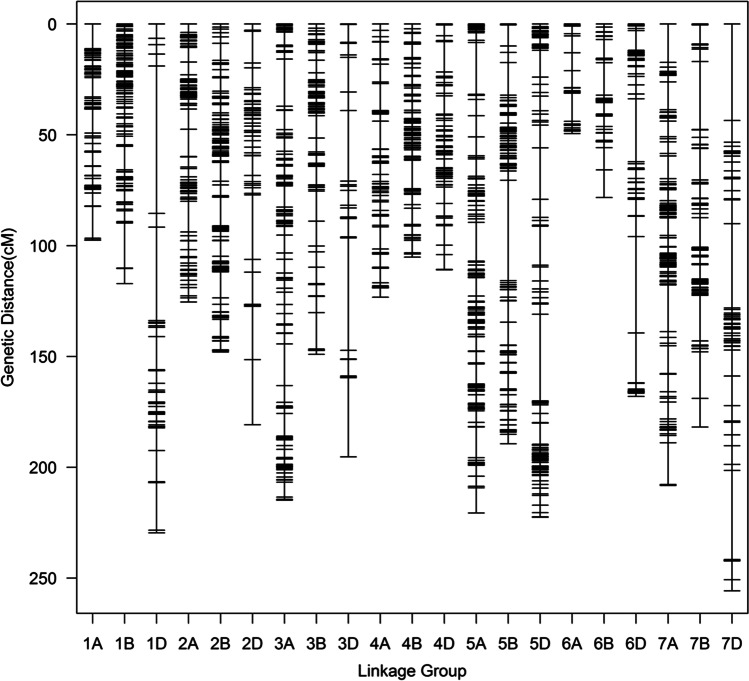
Genetic map of the 228 RIL population obtained from M96-5 × GX3

Preliminary localization was performed using IciMapping 4.1 software. These results showed that an important QTL was located at the end of chromosome 2AS with an estimated LOD value of 40. We therefore selected 22 pairs of SSR primers at the distal region of chromosome 2AS and performed PCR amplification on the parental lines (GX3 and M96-5), a disease-resistant pool (B_R_), and a susceptible pool (B_S_). Four markers (*cfd36*, *wmc382*, *barc124*, and *wmc296*) showed successful amplification consistent between the resistant parent and the resistant pool, and then, there are polymorphisms in the resistant pool and the sensitive pool. The four SSR markers were then used in combination with the 1543 SNPs obtained from genotyping data to analyze the QTL for resistance to wheat stripe rust at the adult plant stage. The final genetic map included 21 linkage groups corresponding to the 21 chromosomes (Fig. 3 and Table 3). The total length of the genetic map was 3371.20 cM, with a mean marker/bin interval of 0.46 cM. Chromosome 7D was the longest (255.73 cM, 0.23 markers/cM), and chromosome 6A was the shortest (49.45 cM, 0.47 markers/cM). Chromosome 2A had 91 markers with a genetic map of 125.43 cM (0.73 markers/cM).

**Table 3:** SNPs statistics of the distribution and density on 21 chromosomes

Chromosome	Length (cM)	No. of markers	Marker density (cM/locus)
1A	97.55	61	0.63
1B	117.13	103	0.88
1D	229.57	41	0.18
2A	125.43	91	0.73
2B	147.97	112	0.76
2D	180.82	47	0.26
3A	214.86	112	0.52
3B	149.02	77	0.52
3D	195.30	26	0.13
4A	123.25	60	0.49
4B	105.15	89	0.85
4D	110.91	61	0.55
5A	220.64	124	0.56
5B	189.42	99	0.52
5D	222.58	95	0.43
6A	49.45	23	0.47
6B	78.26	35	0.45
6D	168.03	54	0.32
7A	208.26	111	0.53
7B	181.87	68	0.37
7D	255.73	58	0.23
Total	3371.20	1547	0.46

In the wheat genome, the B genome was found to have the highest number of markers (583, 37.69%), whereas the D genome had the fewest (382, 24.69%). Among the seven homologous groups of wheat, the fifth homology group had the highest number of SNPs (318, 20.56%), while the sixth homology group had the fewest (112, 7.24%); for the 21 chromosomes in wheat, there was an average of 75 markers for each chromosome. Chromosome 5A harbored the highest number of markers (124, 8.02%), while chromosome 6A had the fewest (23, 1.49%) (Table 4).

**Table 4:** Distribution of polymorphic SNPs on the whole genome

	Group 1	Group 2	Group 3	Group 4	Group 5	Group 6	Group 7	Total
A genome	61	91	112	60	124	23	111	582
B genome	103	112	77	89	99	35	68	583
D genome	41	47	26	61	95	54	58	382
Total	205	250	215	210	318	112	237	1547

### Mapping the QTL

**Fig. 4:**
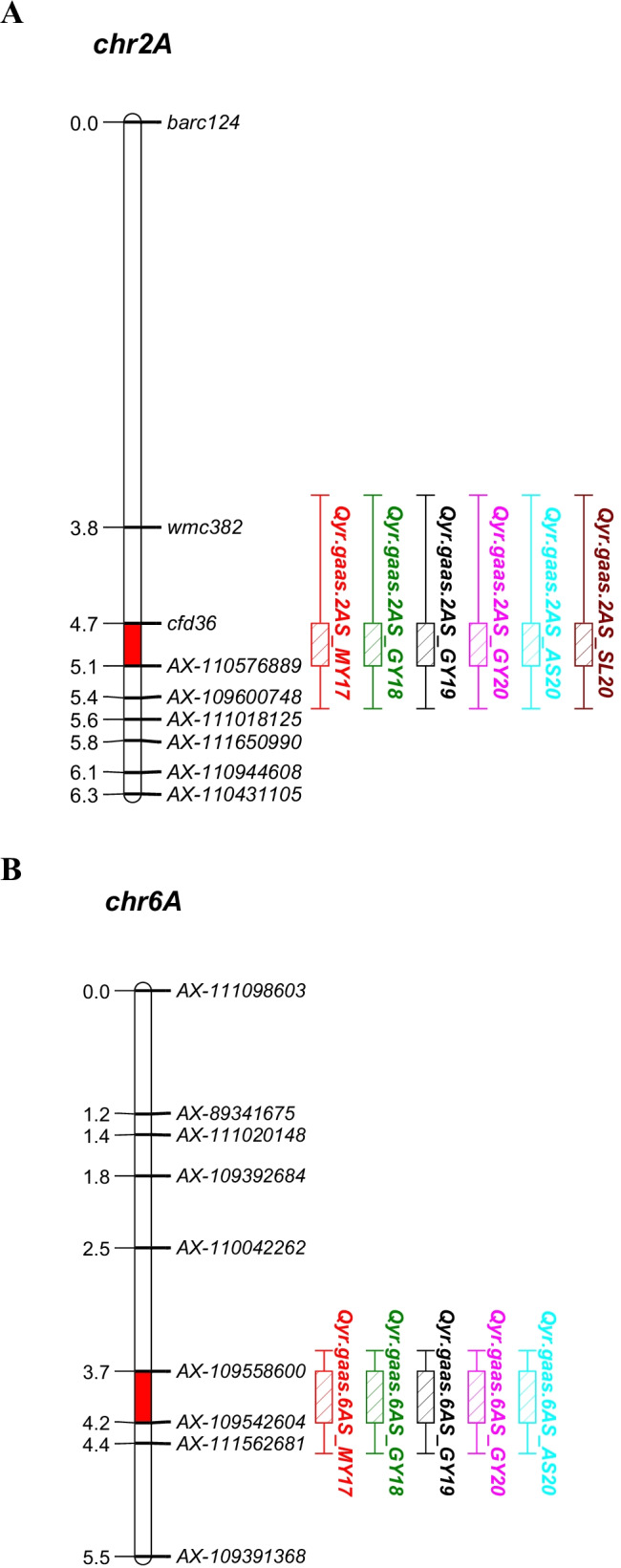
Graphical displays of genetic positions of major QTL for strip rust resistance across six environments for M96-5 × GX3 population, only two chromosomes shown detected two crucial QTL. *Qyr.gaas.2A* on chromosomes 2AS (**A**) and *Qyr.gaas.6A* on chromosome 6AL (**B**) were detected in six and five environments, respectively. Different colors represent different environments: red, 2017 Mianyang; green, 2018 Guiyang; black, 2019 Guiyang; rose, 2020 Guiyang; light blue, 2020 Anshun; brown, 2020 Shuangliu

A total of 19 QTL for resistance to wheat stripe rust were located on 12 chromosomes, including 1B (2), 1D (2), 2A (2), 2B (2), 2D (1), 4B (2), 4D (1), 5A (3), 5B (1), 6A (1), 6B (1), and 7B (1), of which two stable QTL on chromosome 2AS (*Qyr.gaas.2A*) and 6AL (*Qyr.gaas.6A*) were detected in six and five different environments, respectively (Table 5, Fig. 4). An important QTL (*Qyr.gaas.2A*) was located at the position of 5 cM on chromosome 2AS. The 6-year LOD values ranged from 8.01 to 44.61 with confidence intervals of *cfd36* ~ *AX-110576889* (3.5–5.5 cM), which explains the high phenotypic variation of 45.52%. *Qyr.gaas.6A* was located at 4 cM of the distal chromosome 6AL with a confidence interval of *AX-109558600* ~ *AX-109542604* (3.5–4.5 cM), accounting for a phenotypic variation of 3.27–21.73%. In addition, a QTL (*Qyr.gaas.6BL*) was identified in three different environments (Mianyang in 2017 and Guiyang in 2018 and 2019), with a confidence interval of *AX-109408478* ~ *AX-110409180* (54.5–60.5 cM) and a phenotypic variation of 2.01–6.66%. The additive effect of the above three QTL was negative, indicating that their disease resistance originated from GX3. Moreover, another QTL (*Qyr.gaas.2D*) was identified in three environments (Mianyang in 2017, Guiyang in 2018, and Anshun in 2020), with a phenotypic variation of 2.56–3.55%. The additive effects were positive, indicating that its disease resistance originated from the other parental line, M96-5.

**Table 5:** Summary of stripe rust resistance QTL detected in the M96-5 × GX3 RIL population across six environments by ICIM

Environments	QTLs	Marker interval	Position/cM	Confidence interval/cM	Genetic interval/cM	Physical interval/Mb	Physical location/Mb	LOD	PVE (%)	Add
2017 MY	*Qyr.gaas.1B*	*AX-111572690* ~ *AX-94847267*	1	*0* ~ *1.5*	0.69	3.58	22.15 ~ 25.73	3.04	1.89	− 4.91
	*Qyr.gaas.2A*	*cfd36* ~ *AX-110576889*	5	*3.5* ~ *5.5*	0.46	15.22	16.63 ~ 31.85	44.61	45.52	− 4.48
	*Qyr.gaas.2B*	*AX-109528193* ~ *AX-110426897*	136	*132.5* ~ *141.5*	7.76	6.89	33.54 ~ 40.43	2.7	1.99	− 5.01
	*Qyr.gaas.2D*	*AX-110519154* ~ *AX-111418246*	32	*31.5* ~ *34.5*	1.39	4.91	508.19 ~ 513.10	4.66	2.97	6.09
	*Qyr.gaas.4B*	*AX-111556599* ~ *AX-110016820*	6	*4.5* ~ *8.5*	3.95	2.6	657.99 ~ 660.59	4.12	2.79	− 5.91
	*Qyr.gaas.6A*	*AX-109558600* ~ *AX-109542604*	4	*3.5* ~ *4.5*	0.46	0.78	609.11 ~ 609.89	5.12	3.27	− 6.4
	*Qyr.gaas.6B*	*AX-109408478* ~ *AX-110409180*	56	*54.5* ~ *60.5*	10.02	69.9	48.13 ~ 118.03	3.25	2.01	− 5.02
2018 GY	*Qyr.gaas.1D*	*AX-110147378* ~ *AX-109183884*	54	*53.5* ~ *54.5*	0.69	0.43	11.18 ~ 11.61	2.94	1.69	− 5.01
	*Qyr.gaas.2A*	*cfd36* ~ *AX-110576889*	5	*3.5* ~ *5.5*	0.46	15.22	16.63 ~ 31.85	41.93	38.17	− 24.23
	*Qyr.gaas.2B.1*	*AX-108837623* ~ *AX-110359903*	46	*44.5* ~ *46.5*	0.93	7.46	698.22 ~ 705.68	3.22	1.9	− 5.28
	*Qyr.gaas.2D*	*AX-111418246* ~ *AX-110833961*	33	*31.5* ~ *34.5*	1.85	5.09	513.10 ~ 518.18	4.34	2.59	6.18
	*Qyr.gaas.4B.1*	*AX-109410422* ~ *AX-110036160*	0	*0* ~ *2.5*	2.16	7.4	663.04 ~ 670.44	4.45	2.57	− 6.16
	*Qyr.gaas.5A*	*AX-109471543* ~ *AX-110616505*	135	*134.5* ~ *136.5*	0.22	0.24	451.88 ~ 452.12	3.29	1.88	5.25
	*Qyr.gaas.6A*	*AX-109558600* ~ *AX-109542604*	4	*3.5* ~ *4.5*	0.46	0.78	609.11 ~ 609.89	26.27	19.11	− 16.8
	*Qyr.gaas.6B*	*AX-109408478* ~ *AX-110409180*	56	*54.5* ~ *60.5*	10.02	69.9	48.13 ~ 118.03	4.67	2.7	− 6.31
2019 GY	*Qyr.gaas.1D.1*	*AX-94444445* ~ *AX-95126907*	62	*59.5* ~ *63.5*	5.67	1.09	9.28 ~ 10.37	8.55	9.49	− 11.03
	*Qyr.gaas.2A*	*cfd36* ~ *AX-110576889*	5	*3.5* ~ *5.5*	0.46	15.22	16.63 ~ 31.85	8.01	8.08	− 10.3
	*Qyr.gaas.2A.1*	*AX-110538140* ~ *AX-110155009*	48	*43.5* ~ *54.5*	12.23	54.06	622.40 ~ 676.46	4.3	4.35	− 7.44
	*Qyr.gaas.4D*	*AX-111688098* ~ *AX-110768844*	0	*0* ~ *0.5*	0.23	0.13	3.59 ~ 3.72	4.64	4.49	7.56
	*Qyr.gaas.6A*	*AX-109558600* ~ *AX-109542604*	4	*3.5* ~ *4.5*	0.46	0.78	609.11 ~ 609.89	19.16	21.73	− 16.73
	*Qyr.gaas.6B*	*AX-109408478* ~ *AX-110409180*	56	*54.5* ~ *60.5*	10.02	69.9	48.13 ~ 118.03	6.72	6.66	− 9.21
2020 GY	*Qyr.gaas.2A*	*cfd36* ~ *AX-110576889*	5	*3.5* ~ *5.5*	0.46	15.22	16.63 ~ 31.85	16.53	22.36	− 15.54
	*Qyr.gaas.5A.1*	*AX-109019768* ~ *AX-108808620*	209	*204.5* ~ *209.5*	0.71	1.3	4.95 ~ 6.25	2.68	3.13	− 5.71
	*Qyr.gaas.6A*	*AX-109558600* ~ *AX-109542604*	4	*3.5* ~ *4.5*	0.46	0.78	609.11 ~ 609.89	21.69	31.3	− 18.06
2020 AS	*Qyr.gaas.1B.1*	*AX-108745931* ~ *AX-110017315*	83	*81.5* ~ *84.5*	2.15	0.22	667.01 ~ 667.23	3.86	3.7	− 7.18
	*Qyr.gaas.2A*	*cfd36* ~ *AX-110576889*	5	*3.5* ~ *5.5*	0.46	15.22	16.63 ~ 31.85	17.88	19.2	− 16.66
	*Qyr.gaas.2D*	*AX-109229475* ~ *AX-110519154*	31	*29.5* ~ *32.5*	1.88	7.52	500.67 ~ 508.19	3.91	3.55	7.01
	*Qyr.gaas.6A*	*AX-109558600* ~ *AX-109542604*	4	*3.5* ~ *4.5*	0.46	0.78	609.11 ~ 609.89	22.84	25.87	− 18.98
	*Qyr.gaas.7B*	*AX-109352027* ~ *AX-109988869*	13	*8.5* ~ *16*	8.55	16.2	725.20 ~ 741.40	2.59	2.64	6.08
2020 SL	*Qyr.gaas.1B.1*	*AX-110017315* ~ *AX-108726041*	85	*84.5* ~ *88.5*	0.7	0.38	667.23 ~ 667.60	4.29	5.08	− 8.73
	*Qyr.gaas.2A*	*cfd36* ~ *AX-110576889*	5	*3.5* ~ *5.5*	0.46	15.22	16.63 ~ 31.85	15.5	20.68	− 17.96
	*Qyr.gaas.5A.2*	*AX-110169414* ~ *AX-111590180*	73	*70.5* ~ *73.5*	1.42	2.2	573.07 ~ 575.26	3.58	4.25	7.97
	*Qyr.gaas.5B*	*AX-110689592* ~ *AX-109479506*	73	*69.5* ~ *99.5*	4.31	5.83	685.07 ~ 690.89	4.98	6.22	− 9.66

*LOD* logarithm of odds score, *PVE* percentage of phenotypic variance explained by individual QTL, *Add* additive effect of the resistance allele. Positive additive effect indicates that increased resistance to disease was contributed by M96-5 allele; negative by GX3 allele

**Fig. 5:**
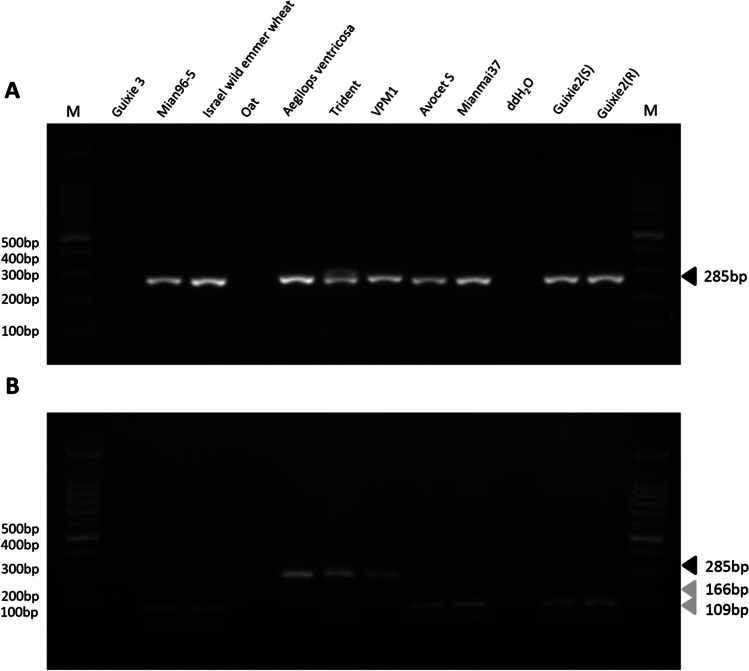
Distinguishing between *Qyr.gaas.2A* and *Yr17* gene by using the specific CAPS marker *URIC/LN2*. PCR fragments were amplified with primers *URIC–LN2* (**A**) followed by *DpnII* digestion (**B**). “M” indicates the molecular markers (100-bp ladder). The black arrowhead indicates target band (285 bp). The gray arrowheads indicate *DpnII* digested fragments (166 and 109 bp)

The specific CAPS marker *URIC/LN2* of *Yr17* (Helguera et al. 2003) was used to determine the differences between parental varieties and their relatives. Our results showed that target bands could not be amplified in the GX3 line (Fig. 5).

### Candidate gene prediction

According to the physical locations of *Qyr.gaas.2A* (*cfd36* ~ *AX-110576889*, 15.22 Mb) and *Qyr.gaas.6A* (*AX-109558600* ~ *AX-109542604*, 0.78 Mb), the sequences located within the interval of two QTL were searched in Triticeae Multi-omics Center to identify the wheat gene ID, annotation, and corresponding sequence. The results identified 620 and 61 segments at the confidence intervals of *Qyr.gaas.2A* and *Qyr.gaas.6A*, respectively (Table S2 and Table S3). The two intervals contained potentially functional genes such as nucleotide binding site-leucine-rich repeat (NBS-LRR), a disease resistance protein, F-box protein, or part of the gene structure directly or indirectly involved in plant disease resistance (Table 6). Thirteen fragments related to disease resistance were clustered on a region of the 2AS chromosome spanning from 17,411,781 bp to 17,601,016 bp, including 10 NBS-LRR (*TraesCS2A01G043500.1*, *TraesCS2A01G037200LC.1*, *TraesCS2A01G043900.1*, *TraesCS2A01-G044000.1*, *TraesCS2A01G044100.1*, *TraesCS2A01G044200.1*, *TraesCS2A01G044300.1*, *TraesCS2A01G044300.2*, *TraesCS2A01G044400.1*, *and TraesCS2A01G044500.1*) and 3 WRKY transcription factors (*TraesCS2A01G043600.1*, *TraesCS2A01G043700.1*, *and TraesCS2A01G043800.1*), while 11 fragments related to disease resistance were clustered on a region of the 6AL chromosome spanning from 609,635,388 bp to 609,796,663 bp, including 8 LRR (*TraesCS6A01G600700LC.1*, *TraesCS6A01G600800LC.1*, *TraesCS6A01G400600.1*, *TraesCS6A01G601000LC.1*, *TraesCS6A01G400700.1*, *TraesCS6A01G601200LC.1*, *TraesCS6A01G400800.1*, *and TraesCS6A01G400800.2*), 2 NBS-LLR (*TraesCS6A01G400900.1*, *TraesCS6A01G401000.1*), and 1 TIR-NBS-LRR (*TraesCS6A01G401100.1*).

**Table 6:** The significant SNPs variations on chromosome 2AS and 6AL

Chrom	Annotation of SNP	Number of SNPs
2AS	Disease resistance protein (NBS-LRR disease resistance protein)	35
	Retrotransposon protein	28
	Retrovirus-related Pol polyprotein from transposon TNT 1–94	19
	F-box protein	15
	Cysteine protease	14
	Proline-rich protein	13
	RNA-directed DNA polymerase	13
	Glycosyltransferase	11
	Receptor kinase	11
	Protein kinase	10
	Transposon protein	10
	Protein FAR1-RELATED SEQUENCE 5	9
	Cytochrome P450	8
	Dirigent protein	8
	Leucine-rich repeat receptor-like protein kinase family protein	7
	Phosphoglycerate mutase-like protein	7
	2-oxoglutarate (2OG) and Fe(II)-dependent oxygenase superfamily protein	6
	Serine/threonine-protein kinase	6
	Zinc finger protein	6
	FAD-binding Berberine family protein	5
6AL	Leucine-rich repeat receptor-like protein kinase family protein	10
	Receptor kinase	5
	F-box protein	4
	Retrotransposon protein	4
	Disease resistance protein(NBS-LRR disease resistance protein)	4

## Discussion

### Wheat stripe rust and phenotypic data

Until 2016, China had officially classified 34 stripe rust races (CYR1–CYR34) and more than 40 pathogenic types (Chen et al. 2014). Since the discovery of a new pathogenic type, V26 in 2009, this pathogenic group has been continuously mutating and expanding. Its aggressive profile has expanded from an initial infection of 12 to 18 Chinese identified hosts, and a survey showed that the frequency of the three pathogenic groups of CYR32, CYR33, and V26 has since exceeded 70% (Zhang et al. 2015). In 2016, the pathogenic group (V26) of Guinong 22 was officially named CYR34, and this has led to an increase in its investigation within the field of wheat research in China.

The Sichuan Basin is a common source of new races of wheat stripe rust in China. Since the pathogenic group (V26) of Guinong 22 was first identified in Sichuan in 2009, it has gradually increased to become the dominant pathogenic group (Xu et al. 2016). At present, there are three main pathogenic groups of stripe rust in Guizhou, namely, HyG pathogenic group, the SuG pathogenic group, and Guinong 22 pathogenic group. Among them, the frequencies of CYR32, CYR33, and CYR34 are 57.14%, 5.71%, and 4.29%, respectively (Chen et al. 2016b). When analyzing the different field phenotypes of RILs between 2017 and 2020, significantly more susceptible varieties were found to originate in Sichuan compared to Guizhou. This difference is mainly due to the different epidemic races of stripe rust found in these two regions. Specifically, CYR32 and CYR33 are found mainly in Guizhou, while CYR34 is predominantly found in Sichuan (Cheng et al. 2020a). We have no evidence that there is a direct correlation between precipitation and disease severity. According to meteorological data, the average precipitation in Guizhou is more than that in Sichuan, but the severity of stripe rust in Sichuan is more serious than that in Guizhou. Therefore, the severity of stripe rust should depend on the combined effects of local spring temperature, humidity, epidemic races, and other factors.

In addition, different varieties carry multiple resistance genes and therefore show different degrees of resistance, although none are completely immune. For each physiological race, selection pressure is greatly reduced, as the host and the pathogen are in a coexisting state, and it is therefore unlikely that new mutations will develop in the pathogen. Due to this phenomenon, the resistance of slow-rust varieties is low, and the resistance in specialized varieties is stronger (Yuan et al. 1995).

### Genetic linkage map

Wheat 55 K SNP array with 53,063 tags was almost evenly distributed across all the chromosomes (Sun et al. 2020). Theoretically, the number of wheat polymorphism markers should be B genome > A genome > D genome, because the wheat D genome has lowest genetic diversity (Marcussen et al. 2014). Our genetic map showed that the least number of polymorphic markers was D genome, which was similar to previous studies (Li et al. 2021; Xiong et al. 2021).

The number of markers on chromosome 6A was the least, and similar result was previously reported (Ren et al. 2018). The possible reason was that the parental lines (Guixie 3 and M96-5) carried *Pm21* gene on the 6VS/6AL translocation lines (Chen et al. 2018; Cheng et al. 2020b), resulting in fewer polymorphic markers on chromosome 6A.

### QTL mapping

In general, QTL that exert a larger effect on phenotype tend to be more stably expressed and are more easily detected within different environments. QTL that exert a smaller effect tend to be more influenced by genetic background and the external environment (Ma et al. 2019; Rehman et al. 2020). These can therefore be more difficult to detect under certain environmental conditions (Li et al. 2010). In this study, two stable QTL were identified on the chromosomes 2AS and 6AL.

At the distal of chromosome 2AS, a significant stable QTL (*Qyr.gaas.2A*) was detected at the interval of *cfd36* ~ *AX-110576889* (16.63 ~ 31.85 Mb) in all six environments assessed in this study. Five genes for the resistance of stripe rust were identified on chromosome 2AS, *Yr17* (*XcMWG682–XksuH9-2A*, 3.96 Mb null), *Yr56* (*Xbarc212–Xgwm512*, 5.23–12.17 Mb), *YrR61* (*Xbarc124–Xgwm359*, 5.23–28.20 Mb), *Yr69* (*X2AS33–Xmag3807*), and *YrZM175* (*Xwmc382–Xgwm636*, 2.32–4.98 Mb). Through pedigree analysis, we found that *YrR61* was derived from the American soft red wheat Pioneer 26R61 (Hao et al. 2011) and *Yr56* from the durum wheat, Wollaro (Bansal et al. 2014). Both of the identified genes are known to promote adult resistance. *Yr69* (Hou et al. 2016) and *YrZM175* are known to be genes that promote seedling resistance in the artificial wheat introgression line, CH7086, and common wheat variety, Zhongmai 175 (Lu et al. 2016), respectively. *Yr17*, derived from *Ae. ventricosa*, is also a seedling-resistant gene (Bariana and McIntosh 1993). The above genes have loss disease resistance to CYR34 race according to previous report (Zeng et al. 2015). However, our field investigations showed that VPMI (the carrier line of *Yr17* gene) present medium resistance to the pathogenic group of Guinong 22 in Guiyang. Moreover, *Yr17* had the closest position to *Qyr.gaas.2A*. The allelism tests will perform to cross between GX3 and the carrier line of *Yr17*, VPMI in the future. Furthermore, 13 QTL have been previously reported on chromosome 2AS (Bulli et al. 2016), of which eight are adjacent to or overlapping with *Qyr.gaas.2A*. GX3 is different from the parental lines of these disease-resistant QTL, so we therefore speculate that *Qyr.gaas.2A* in GX3 is inconsistent with the above QTL.

On the 6AL chromosome, *Qyr.gaas.6A* (*AX-109558600* ~ *AX-109542604*) is located in the interval spanning 3.5 ~ 4.5 cM (609.11–609.89 Mb). *YrLM168*, derived from Chuannong 16, was mapped in the interval of *wmc59*-*wmc145* on chromosome 6AL, which is adjacent to *Qyr.gaas.6A* (Feng et al. 2014; Bulli et al. 2016). Meanwhile, there are four adjacent or overlapping QTL. *QYr-6A_Saar* (*XwPt-7063–Xbarc3*, 62.92–85.28 Mb) was derived from the CIMMYT variety Saar and has two important gene loci (*Lr34/Yr18/Pm38* and *Lr46/Yr29/Pm39*) (Lillemo et al. 2008). *QYrpl.orr-6AL*_Stephens was derived from a commercial wheat variety Stephens, which has been grown in the USA Pacific Northwest for 30 years (Dolores et al. 2012). *QYr-6A_Avocet* (*gwm617*) came from the susceptible parent “Avocet S” (William et al. 2006). *QYrtb.orz-6AL* (*wPt-4229*, 611.41 Mb) was from the hard red winter wheat cultivar “Einstein” from Limagrain, UK (Vazquez et al. 2015). Due to the different sources of the identified parental QTL, *Qyr.gaas.6A* is likely to be inconsistent with the aforementioned QTL.

In our last study about genome-wide association analysis of resistance to strip rust (Cheng et al. 2020a), four SNPs loci on three chromosomal regions were significantly associated with *Pst* resistance at Mianyang in 2014: *Tdurum_contig29087_628* (1BL, 661.63 Mb), *Tdurum_contig 29087_757* (1BL, 661.63 Mb), *TA002369-0369* (4AS, 245.96 Mb), and *wsnp_Ex_c965_1846161* (6AL, 581.75 Mb). We considered that the four SNPs were likely to be associated with new QTLs/genes that confer resistance to race CYR34. In this study, we detected a QTL (*Qyr.gaas.1B.1*, 667.01–667.60 Mb) that was located closely to *Tdurum_contig29087_628* and *Tdurum_contig29087_757*, which may not be associated with *YrExp1* (637.39 Mb) (Lin and Chen 2008). However, no QTL was mapped on chromosome 4A, and *Qyr.gaas.6A* (609.11–609.89 Mb) was inconsistent with the SNP locus *wsnp_Ex_c965_1846161* (6AL, 581.75 Mb) on chromosome 6A.

### Candidate gene prediction

Response to biological stress in plants can often be dependent on a variety of cell receptor proteins. Intracellular receptor proteins are encoded by NBS-LRR disease resistance genes, which have the ability to directly or indirectly recognize effector molecules (effector) released by the pathogen into the cell and trigger a disease resistance response. This response is referred to as effector triggered immunity (Noutoshi et al. 2005) and requires mediation of the transcription factor WRKY. The NBS domain binds to ATP or GTP to play a key role in plant disease resistance.

Currently, the cloned rust genes mainly encode the following three resistance proteins: (I) CC-NBS-LRR, such as stripe rust resistance gene *Yr10* (Liu et al. 2014), *Yr28* (Zhang et al. 2019), leaf rust resistance genes *Lr10* (Feuillet et al. 2003), *Lr21* (Huang et al. 2003), stem rust resistance genes *Sr33* (Periyannan et al. 2013), *Sr35* (Saintenac et al. 2013), (II) WRKY-NBS-LRR, such as *YrU1* gene (Wang et al. 2020), and (III) NBS-LRR, such as *Lr1* (Cloutier et al. 2007).

In the present study, we identify *Qyr.gaas.2A* (*cfd36* ~ *AX-110576889*) and found a structural region (chr2A:17411781_17601016) containing 10 NBS-LRR and 3 WRKY transcription factor near *cfd36* marker (16.63 Mb). We therefore speculated that the structural region in combination with a relatively complete NBS-LRR may represent a novel candidate gene, and these 13 identified segments will be further investigated. We also found the similar candidate gene structure on chromosome 6AL; this area (*AX-109558600* ~ *AX-109542604*, chr6A:609635388_609796663) contained eight LRR, two NBS-LLR, and one TIR-NBS-LRR. We hence considered that one of the 11 candidate segments in this region is most likely to be novel *Yr* gene.

GX3 was derived from wild crossing between wild emmer wheat (*T*. *dicoccoides*) and wild oat (*Avena fatua* L. var. *glabrata pat*) varieties and was subsequently obtained by backcrossing with the common wheat Guinong 22 variety. Field observations spanning many years have shown that wild emmer wheat and Guinong 22 are susceptible to CYR34, while the wild oat is immune. Therefore, we speculate that the major QTL on chromosome 2AS originated from *Avena fatua* L. var. *glabrata*. However, in situ hybridization analysis showed no signal detection in the wild oat variety samples (unpublished data). These findings may be due to the fact that the introgression fragment is too small. These experiments therefore require further validation.

## Conclusions

High-yield and disease-resistant new lines could be bred by crossing GX3 with susceptible varieties possessing excellent agronomic traits. Two stable and reliable QTLs (*Qyr.gaas.2A* and *Qyr.gaas.6A*) were identified from biparental populations, and four closely linked markers could be transformed into KASP or SSR markers for marker-assisted selection breeding. Several resistance genes/QTL has been previously mapped on chromosomes 2AS and 6AL; we hence will perform further research to determine whether they are new disease resistance genes/QTLs and simultaneously increase the density of genetic maps around the two QTLs to clone the underlying gene.

## Supplementary Information

Below is the link to the electronic supplementary material.

Supplementary file1 (XLSX 63 kb)
